# Finger Prosthesis: An Economic and Esthetic Approach

**DOI:** 10.7759/cureus.41989

**Published:** 2023-07-17

**Authors:** Arunoday Kumar, Suchetana Basak, Rajesh S Nongthombam, Babina Chirom, Poukhuan Panmei

**Affiliations:** 1 Dentistry, Regional Institute of Medical Sciences, Imphal, IND; 2 Dentistry, Dayananda Sagar College of Dental Sciences, Bengaluru, IND

**Keywords:** plantar surface, dorsal surface, aesthetic, acrylic resin, maxillofacial material, prosthesis

## Abstract

Fabrication of prosthetic fingers is more of an art than a science. The prosthetic finger must match the missing fingers or digits to such an extent to have a natural aesthetic look for the patient. This would build up the patient's confidence and prevent unwanted attraction by others.

A patient, 47 years old, reported the loss of the middle finger of the right hand. The loss of the right middle finger was till the first phalanx, and it was distorted till the second phalanx. The patient wanted to have a natural look at his hand. Alginate impressions of the patient's amputated finger and the donor's relevant matching fingertip were made. A waxed prosthesis pattern was fabricated with the donor's fingertip. A wax trial was done in the patient, and was flasked. A heat cure acrylic was intrinsically painted with acrylic paint color to match the adjacent finger's skin tone, followed by packing these in the dewaxed mold, processed, trimmed, finished, and polished. This case report describes a method of obtaining or enhancing retention of the prosthetic finger by adopting a customized finger ring on the master cast and also customizing the prosthesis in the clinical rest position to have the aesthetic appearance in the resting condition of the palms and fingers. This method is cheaper and very easy to be adopted for affected people.

## Introduction

Patients who meet with an accident or due to some surgery lose some of the tissues and need restoration for good aesthetics and function. The primary treatment for such cases is surgery, but surgeries cannot rectify all the defects. Some tissue loss or defects can be reformed by prosthetic rehabilitation. Prosthetic rehabilitation is an artificial method to replace or reform any part of the human, like fingers, eyes, ears, etc. [1]. The hand is an important part of our body that helps us perform our day-to-day functions, and fingers are an integral part. Hands without fingers are non-functional as mouth without teeth. It helps us perform various daily functions and gives us an aesthetic appearance. Loss or absence of the finger or part of the finger could be congenital, acquired malformation (traumatic/accidental), or surgical amputation [2]. Losing a finger brings psychological trauma to an individual to appear in public. An aesthetically fabricated finger prosthesis regains back the psychological trauma created due to its loss [3].

Various factors for the success of finger prosthesis depend on how well the treatment planning of the case is done, clinical case presentation, choice of material for primary impression making and pouring of the cast, wax carving of the finger, and material of choice for the fabrication of permanent prosthesis [4]. Retention of the prosthesis and the shade match is challenging for the prosthodontist in various clinical presentations of the amputated finger. Use of titanium implants, adhesives are generally used to enhance the retention of the prosthesis [5].

In this case report, the use of a finger ring was adopted as opted by the patient itself. It was done onto the selectively trimmed stump cast to create uniformly distributed positive pressure around the remaining digits of an amputated finger. Excellent retention was enhanced by relining the tissue surface of the prosthesis using soft relining material. The procedure chosen in this case report can be called a traditional method for fabricating prosthetic fingers. It requires highly skilled professionals and hard work compared to the Computer Aided Designs (CAD) of rapid prototyping and 3D printing to fabricate the prosthetic finger [6]. But, this modern technique is less affordable to the general public.

This case report explains a conventional technique for fabricating prosthetic fingers with increased retention by using finger rings and soft relining material for better comfort at a very economical rate which is affordable by the weaker sections of society.

## Case presentation

**Figure 1:**
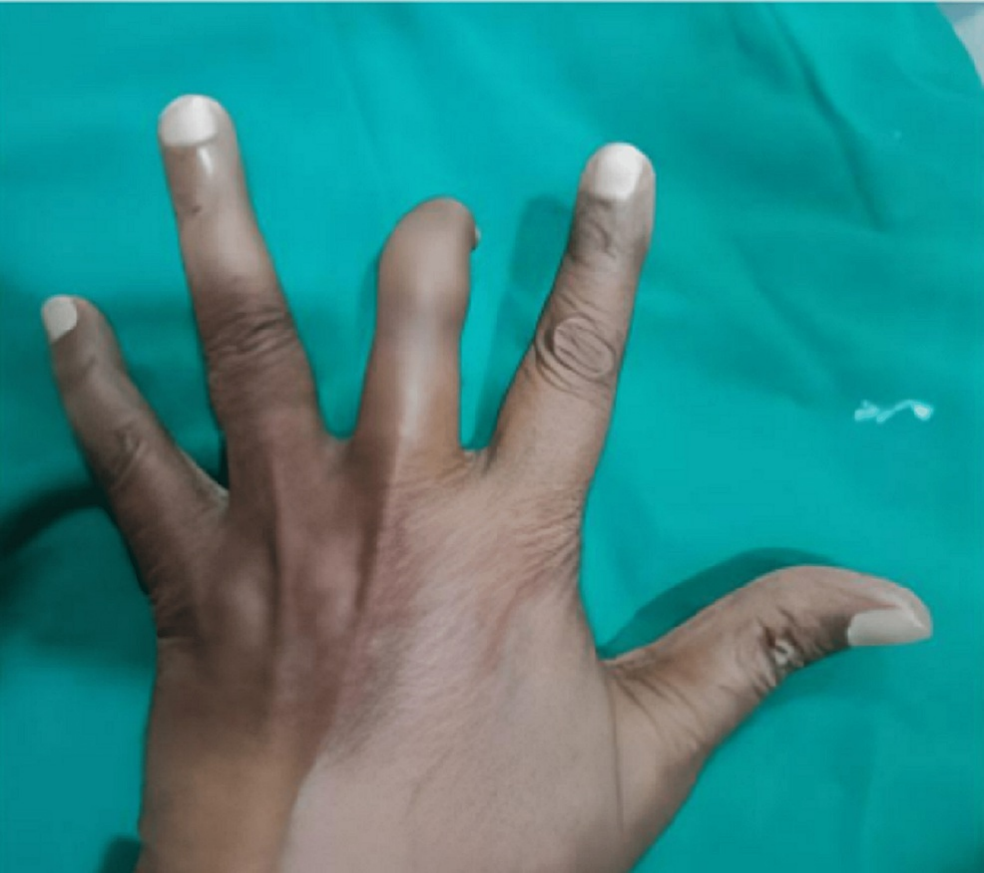
Pre-prosthetic Dorsal surface

**Figure 2:**
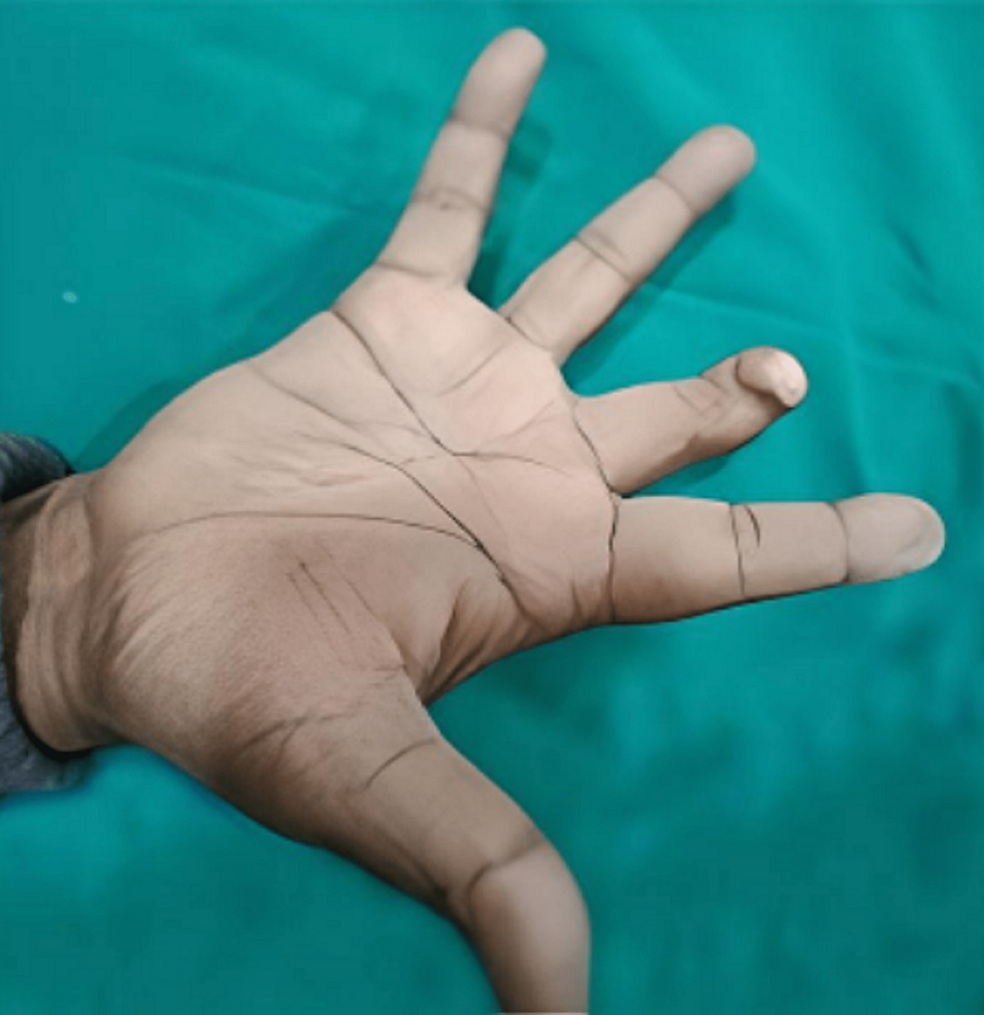
Pre-prosthetic Plantar surface

A 47-year-old male patient visited the Dept of Prosthodontics, Dental College, RIMS, having OPD no 2023/080/000452. A detailed case history revealed the loss of the right middle finger due to surgical removal because of severe infection, leaving deformed digits at its tip for the last five years. Fig 1 and Fig 2 show the dorsal and plantar surface of a pre-prosthetic finger of the patient, respectively.

On careful examination of the surgical site, there was healthy, well-keratinized epithelium which was a clear indication of successful prosthesis fabrication. The patient did not give any history of prosthetically rehabilitated fingers prior. The patient had consented to the prosthesis fabrication of his finger following the ethical norms and conditions.

Treatment

**Figure 3:**
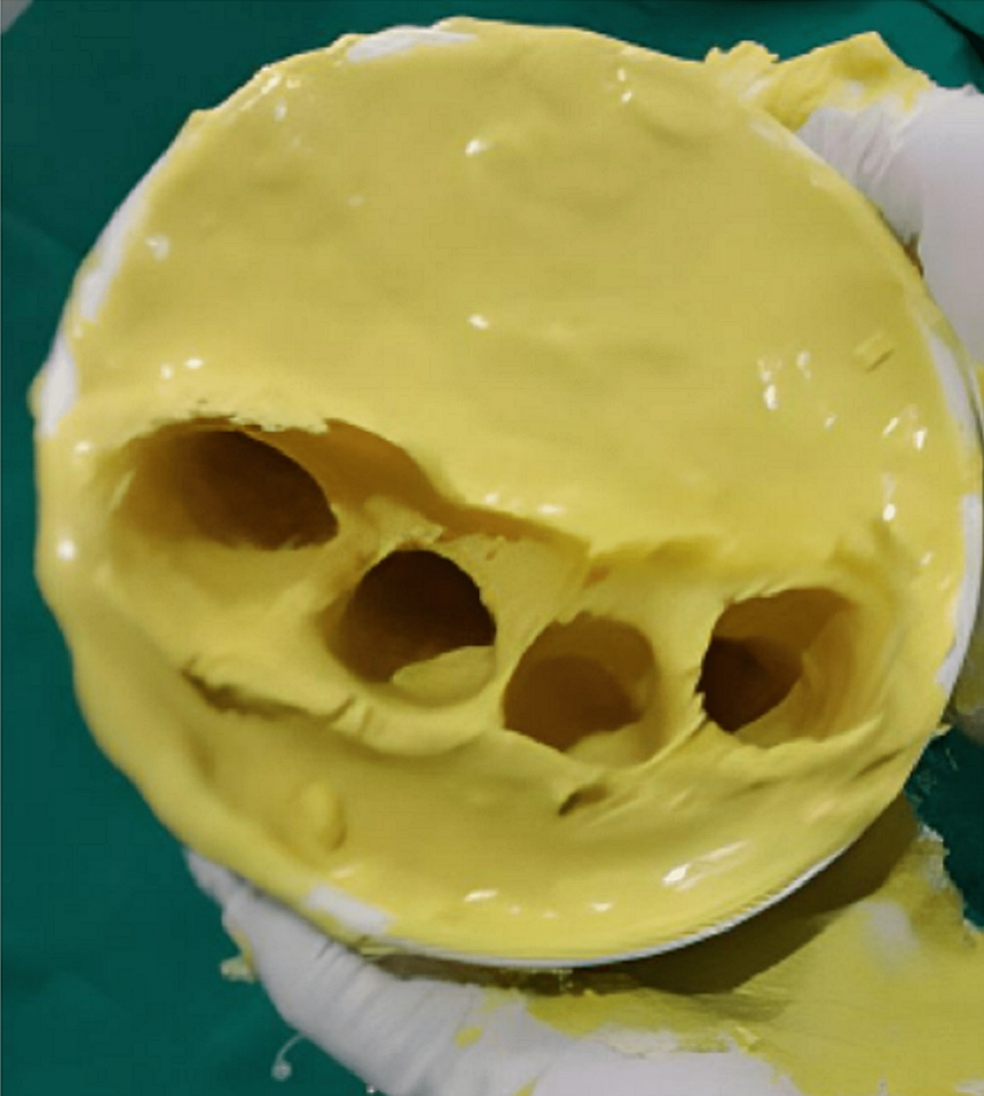
Alginate impression of all four fingers of the right hand, including the amputated finger.

Firstly, the patient was made to wash his hands with the provided antiseptic soap solution and wipe with the cotton. Then, a thin layer of petroleum jelly was applied, followed by a complete alginate impression of all four fingers till the palm was made. Impression of all four fingers, including the amputated one, is made to have a guide of the angulation and curvature of the fingers in the resting condition of the hand, as shown in Fig 3. 

It is poured with type III dental stone (Kalstone. Kalabhai, dental stone class III). The master cast thus obtained is used for fabricating an acrylic finger prosthesis by adapting the finger wax pattern onto the positive replica of the amputated finger.

Procedure for wax pattern fabrication

Another impression of the right middle finger of the donor is made, which is generally from the sibling to have the matching dimension of the finger, and it is poured with the modeling wax. On solidification, it was retrieved, modified, and adapted onto the positive replica of the amputated finger. It was aligned in the same curvature as the other fingers in resting condition. This would give a life-like appearance in natural resting conditions. The wax pattern was further characterized to mimic the skin folds and wrinkles.

**Figure 4:**
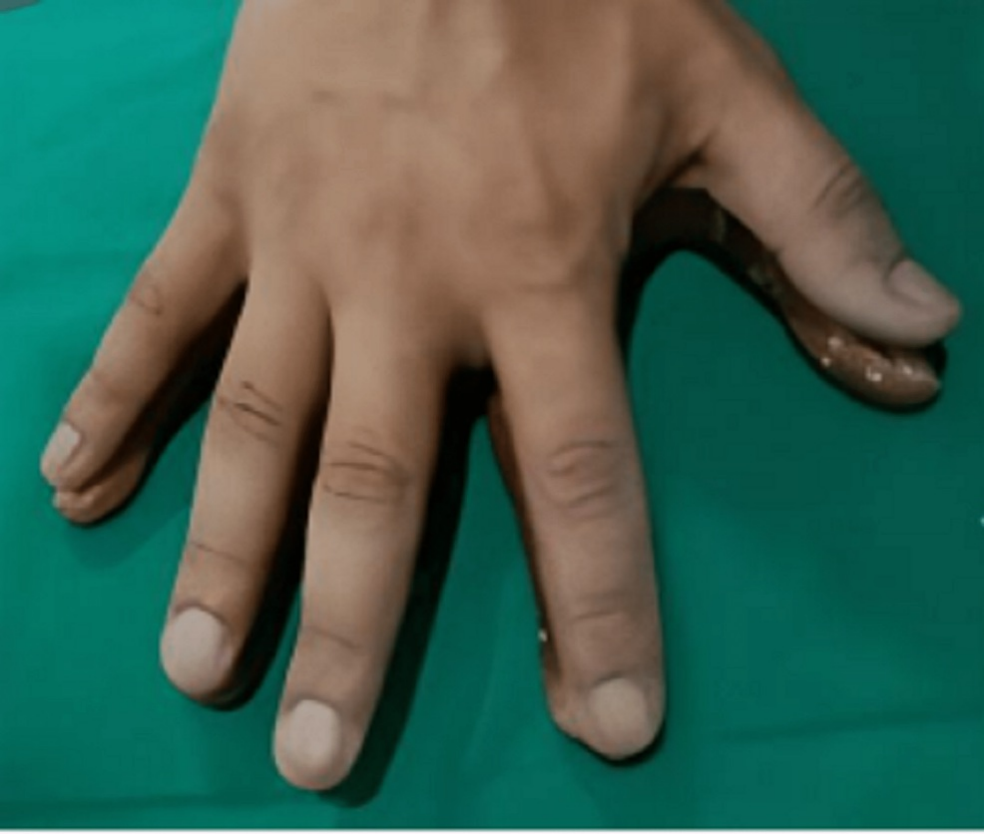
The width and length of the patient’s Phalanxes are matched with the donor.

The distal phalanx was palpated manually to know the diameter of the hard tissue underneath, where the finger ring had to get adapted. The width and length of the patient's Phalanxes are matched with the donor, which is presented in Fig 4.

**Figure 5:**
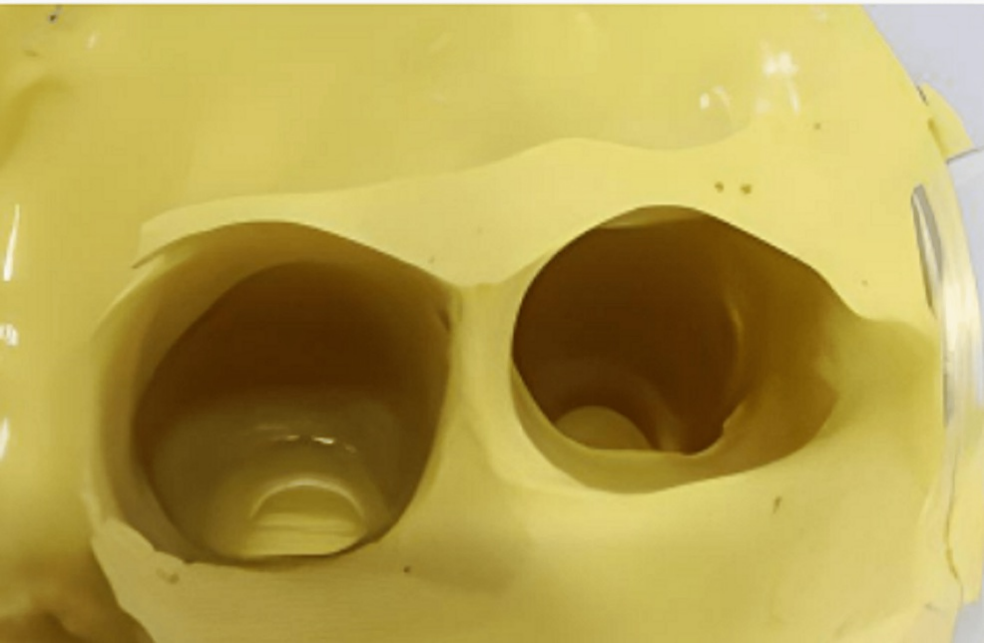
Alginate impression of the relevant right middle finger of the donor

**Figure 6:**
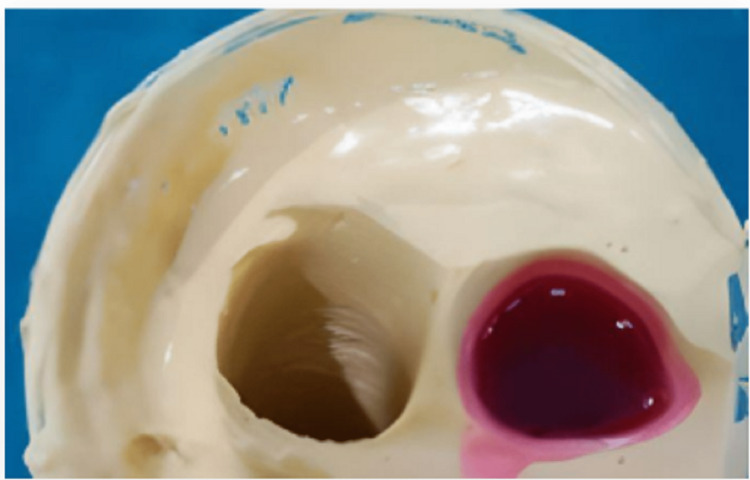
Wax pouring of the impression

Based on the compressibility of the soft tissue, the positive replica of the amputated finger was indexed and trimmed to about 1.5 to 2 mm depth. Trimming was greater in the region of greater compressibility. The position of the finger ring was decided at the meta-carpopharyngeal joint. The trimming was also done in a ring shape having uniform width of 2mm, which matched with the dimension of the finger ring and was attached to it, as presented in Fig 5, and wax pouring of the impression is shown in Fig 6.

**Figure 7:**
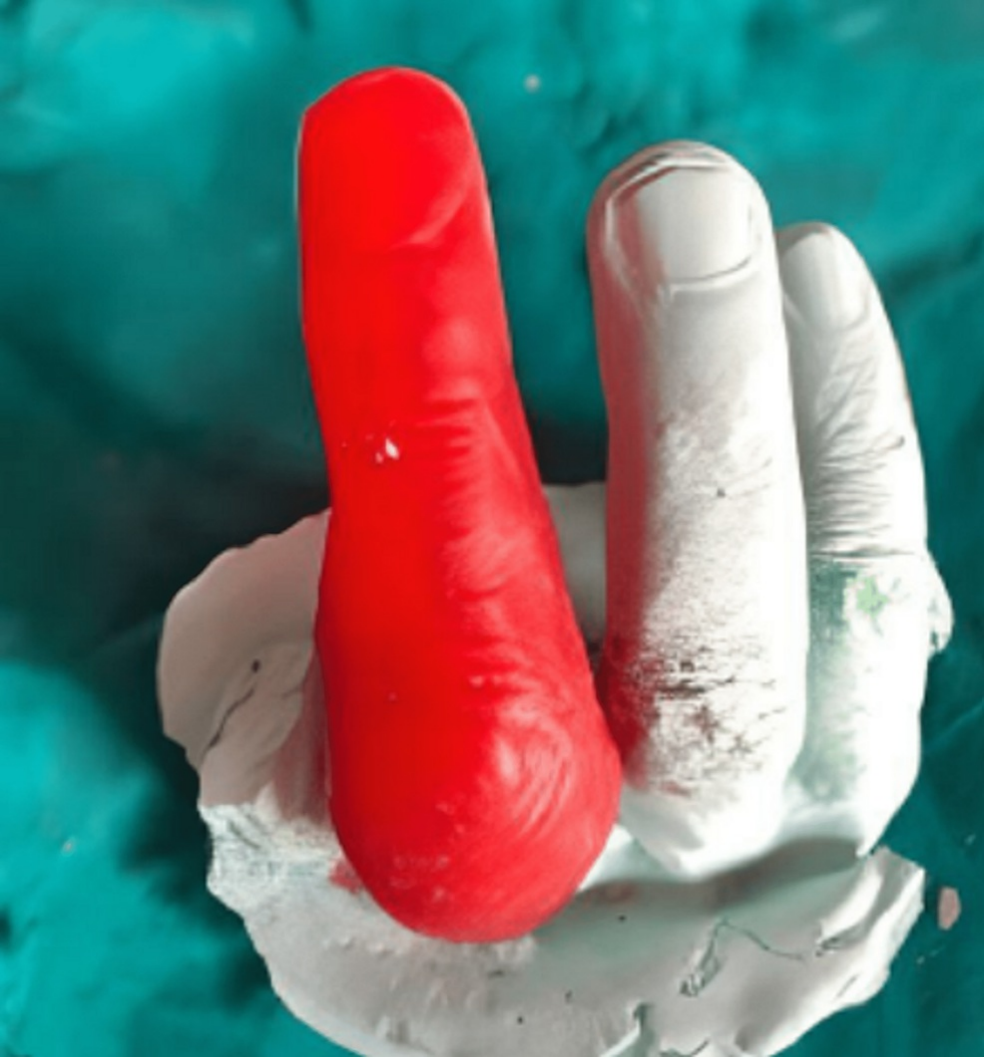
Wax pattern attached to the patient’s amputated finger and matched with the curvature of remaining fingers in resting condition

The wax pattern and the amputated finger cast mold were flaked using type II dental plaster, dewaxed, and packed with characterized secondary acrylic color, which would match the adjacent skin color of the patient's finger. Coloring pigments were added to match the specific color of the folds and wrinkles of the finger. Fig 7 shows the wax pattern attached to the patient's amputated finger and subsequently matched with the curvature of the remaining fingers in resting condition.

**Figure 8:**
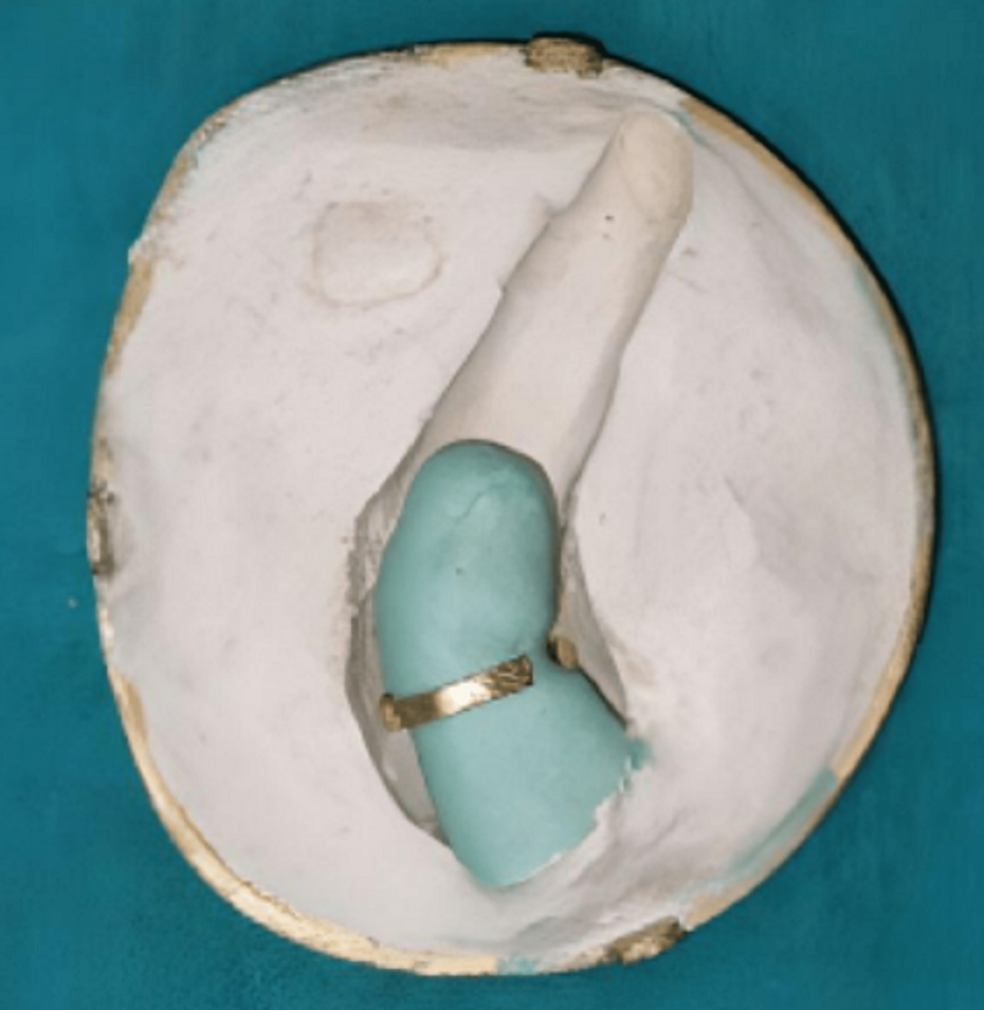
Finger ring attached to the indexed and trimmed amputated finger cast as shown during dewaxing of the mold

Only the wax pattern along the ring placed on the amputated finger cast was flasked, dewaxed, and packed with the characterized heat cure acrylic resin as a maxillofacial material, as shown in Fig 8.

**Figure 9:**
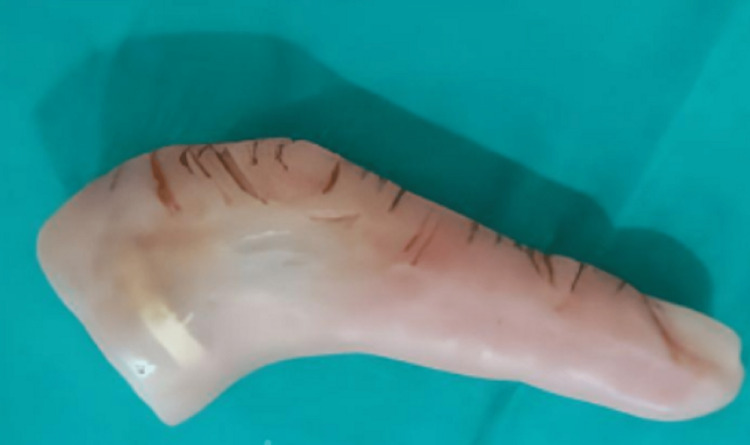
Finished and polished finger prosthesis

The flask was closed under light pressure, transferred to a clamp, left for table curing for half an hour, and followed by processing in the fast curing cycle of heat cure acrylization, i.e., 74^0^C water bath for 2 hours and 100^0^C for 1 hour. After cooling the table for another half an hour, it was deflasked, retrieved, trimmed, and polished to obtain the final finger prosthesis, as shown in Fig 9.

Figure 9 also shows the ring incorporated for good retention, obtained after Packing the mold with characterized secondary color of heat cure acrylic resin, processed, retrieved, trimmed, and polished.

**Figure 10:**
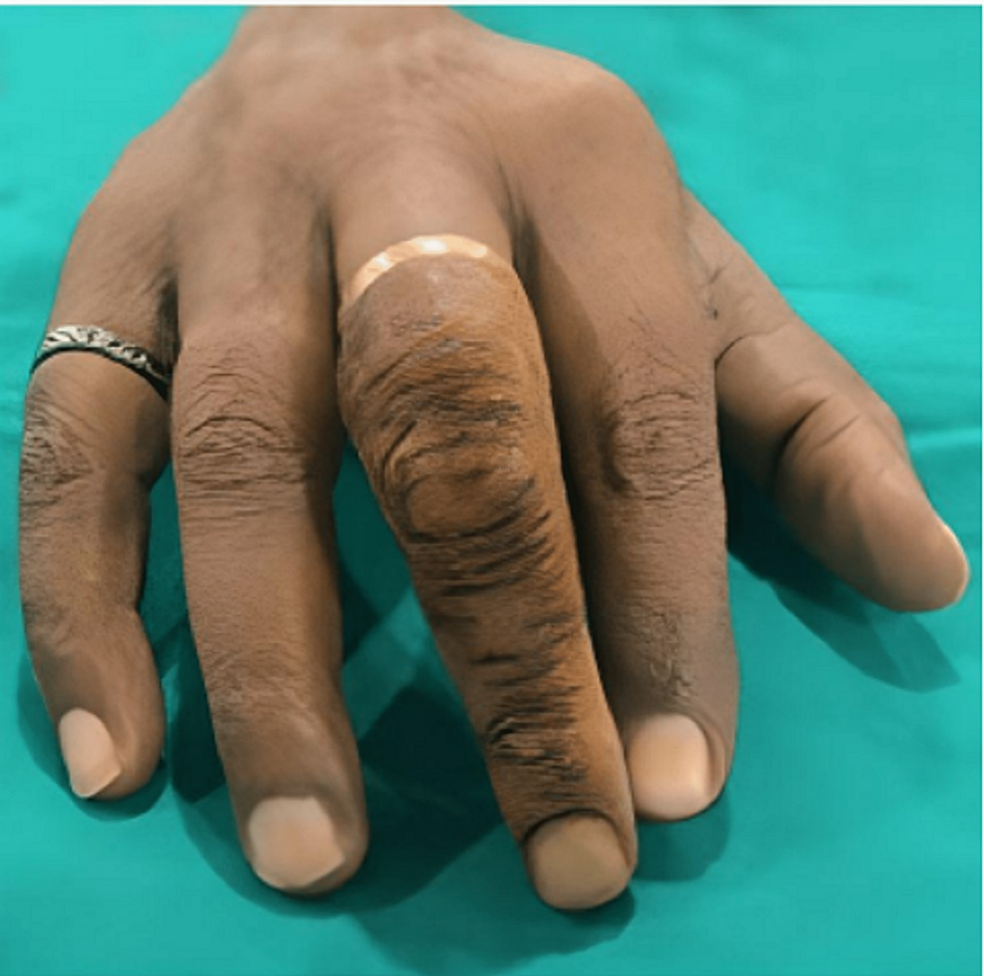
Finished final finger prosthesis Dorsal Surface

**Figure 11:**
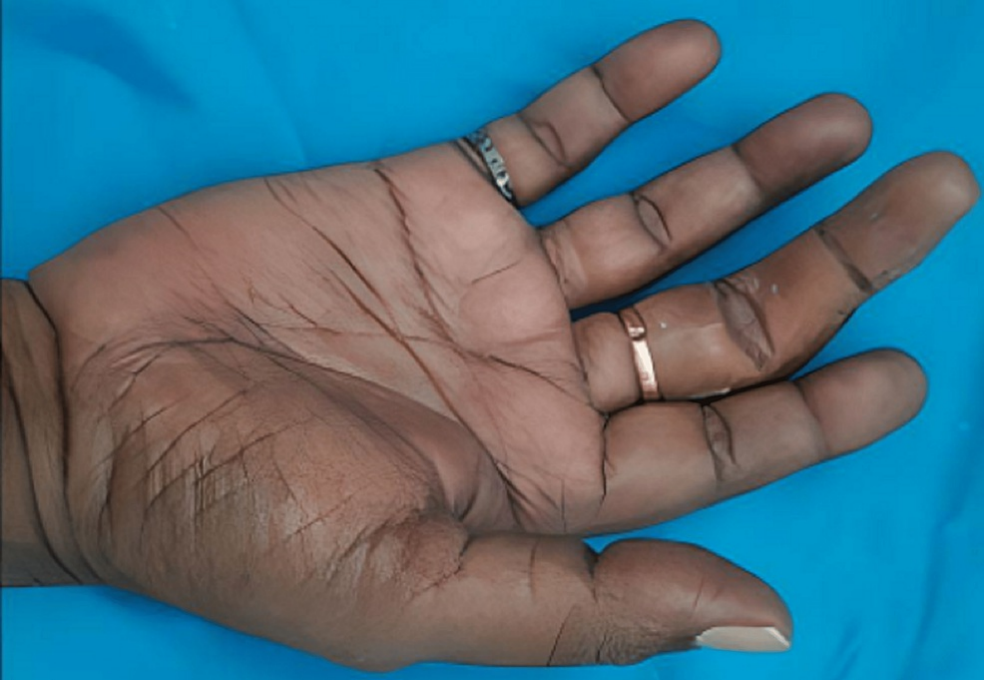
Finished final finger prosthesis Plantar Surface

The prosthesis was color matched with the adjacent skin of the patient. For better color and characterization, the prosthesis was eccentrically stained with extrinsic stains in both dorsal and plantar surfaces under daylight conditions, as shown in Fig 10 and Fig 11, respectively. The nail bed was customized with self-cure acrylic resin and pigmented to match the patient's adjacent nailbed color, as shown in Fig 10.

The patient had good prosthesis retention because the finger ring adapted to the stump cast, and he was happy with the final prosthetic finger. The patient was made aware of the maintenance of the prosthesis by cleaning the skin, using lukewarm water, and irrigating with a soap solution.

## Discussion

The artificial finger can be given in a condition where the remaining parts of the digits or phalanx are available to retain the prosthesis. The artificial finger is planned in conditions where the surgical replantation or reconstruction is impossible, like severe infection or delayed consultation with the surgeons wherein the amputated finger is not preserved as per the recommended protocol, and accidental loss of the finger can not be tracked. Replantation of the digits to reattach the fingers is possible if the patient turns up at the surgical clinic within 4 to 6 hours after injury. The patient reported that after 40 years of digital loss and deformity in this case, so we planned for an artificial finger prosthesis.

The better prognosis of any prosthesis depends on factors like retention, stability, support, esthetics, and patient comfort [7]. These factors are governed by proper treatment planning, impression making, wax carving, and the best material suitable for clinical presentation.

Maxillofacial material

The most commonly used maxillofacial materials are silicones and polymethyl acrylics. Silicones are available in grades I, II, III, and IV and are named implant grade, medical grade, clean grade, and industrial grade, respectively [8]. Out of these grades of available silicones, medical grade, i.e., grade II, is used for the fabrication of maxillofacial prosthesis and is the most commonly used. But because of the high cost of silicone materials, we as a prosthodontist sometimes recommend acrylic prosthesis as a preferred maxillofacial prosthesis material for the finger prosthesis for patients of low socioeconomic status because of its low cost [9]. Heat-cure acrylic resin is preferred over cold-cure acrylic resin because of its low residual monomer and high color stability. Heat cure acrylics are Polymethyl methacrylate as powder and Methylmethacrylate as a monomer [10]. The colors for specific extrinsic skin shades are acrylic-based paints mixed in monomer or chloroform-based solvents. Intrinsic stains commonly used are rayon flocking or fibers, dry earth pigments, etc. We used heat cure acrylic resin material to fabricate finger prostheses as the material is readily available, cheaper, and affordable by the weaker sections of society. Other advantages of this material are that they are durable, color stable, and can be relined and repaired.

We can use acrylic resin material for the fabrication of finger prosthesis when residual phalanges measures more but not less than 2cm where provision for direct fixation is possible. A patient's acceptance of the prosthesis depends on how aesthetic it is and how active grasp it can provide. Retention of the prosthesis can be provided by medical grade adhesives, implants, retentive finger rings, or clasp, which should make positive contact with phalange's hard tissue.

Leow et al. [11] showed optimal trimming in the finger model for good prosthesis retention for distal finger amputation for about 5-7 % circumference reduction. In this case, in the report, we made the circumferential reduction of 1-2 mm depending on the skin's flexibility, and then the finger ring was attached to the stump and finally processed. The final fit of the prosthesis was accessed and defined by relining the prosthesis with the soft relining material.

Recent advances

Recently prosthetic hands are new innovations in prosthetic rehabilitation of hands and fingers, which can actively abduct and adduct with the implementation of various electronic configurations. Cabibihan has successfully worked on fabricating characterized fingers, which can replicate its function using Computer Tomography (CT) data of patients remotely [12-14]. But because of its high cost, the use of this technology to fabricate hands or fingers is limited; still, this method is useful in pandemic conditions like Corona.

## Conclusions

This case report provides economic and esthetic management of amputated fingers by prosthetic rehabilitation. It describes a method of obtaining and enhancing the retention of the prosthetic finger by the use of a customized finger ring on the master cast and also a customized finger prosthesis in the clinical rest position. A prosthetic finger is made using the traditional method of finger fabrication using heat cure acrylic resin. A finger ring and soft relining material are used to increase retention. This method provides a comfortable, lightweight, low-cost, matching skin color, wearable prosthetic finger to the patient in a concise duration.
